# Aortopulmonary collateral arteries: a rare complication after arterial switch operation for transposition of the great arteries

**DOI:** 10.1186/s40792-015-0098-1

**Published:** 2015-10-06

**Authors:** Fumiaki Shikata, Toru Okamura, Takashi Higaki, Masahiro Okura, Chisato Yajima, Ai Kojima, Shunji Uchita, Yuji Sakashita, Kenji Namiguchi, Takumi Yasugi, Hironori Izutani

**Affiliations:** Department of Cardiovascular Surgery, Ehime University, Shitsukawa, Toon, Ehime 791-0295 Japan; Department of Pediatric Cardiology, Ehime University, Toon, Ehime Japan

**Keywords:** Arterial switch operation, Pediatric cardiac surgery

## Abstract

Collateral vascular arteries from the descending aorta to the pulmonary arteries are uncommon after arterial switch operation. Here, we report the case of a baby girl treated with coil embolization for abnormal blood flow from the descending aorta to the pulmonary arteries after arterial switch operation. A baby girl weighing 1324 g was delivered at 32 weeks 4 days of gestation, and she had D-transposition of the great arteries and a ventricular septal defect. She underwent nitrogen inhalation to reduce pulmonary blood flow before arterial switch operation. After the operation, she presented with left heart failure due to the presence of abnormal blood flow from the descending aorta to the pulmonary arteries, and she was successfully treated with coil embolization. After the treatment, her condition improved dramatically, and she was discharged without any complications.

## Background

The outcomes of neonatal cardiac surgery have improved dramatically owing to advancements in surgical techniques and perioperative care [1]. For D-transposition of the great arteries (D-TGA), which needs neonatal cardiac surgery, selection of the surgical strategy mainly depends on the anomalous heart and vascular factors, such as coronary anatomy, ventricular septal defect (VSD) location, and associated vascular malformations [2]. Collateral vascular arteries from the descending aorta to the pulmonary arteries are uncommon after arterial switch operation, and their presence can lead to several critical conditions such as pulmonary bleeding and low cardiac output [3]. Here, we report the case of a baby treated with coil embolization for abnormal blood flow from the descending aorta to the pulmonary arteries after arterial switch operation.

## Case presentation

**Fig. 1:**
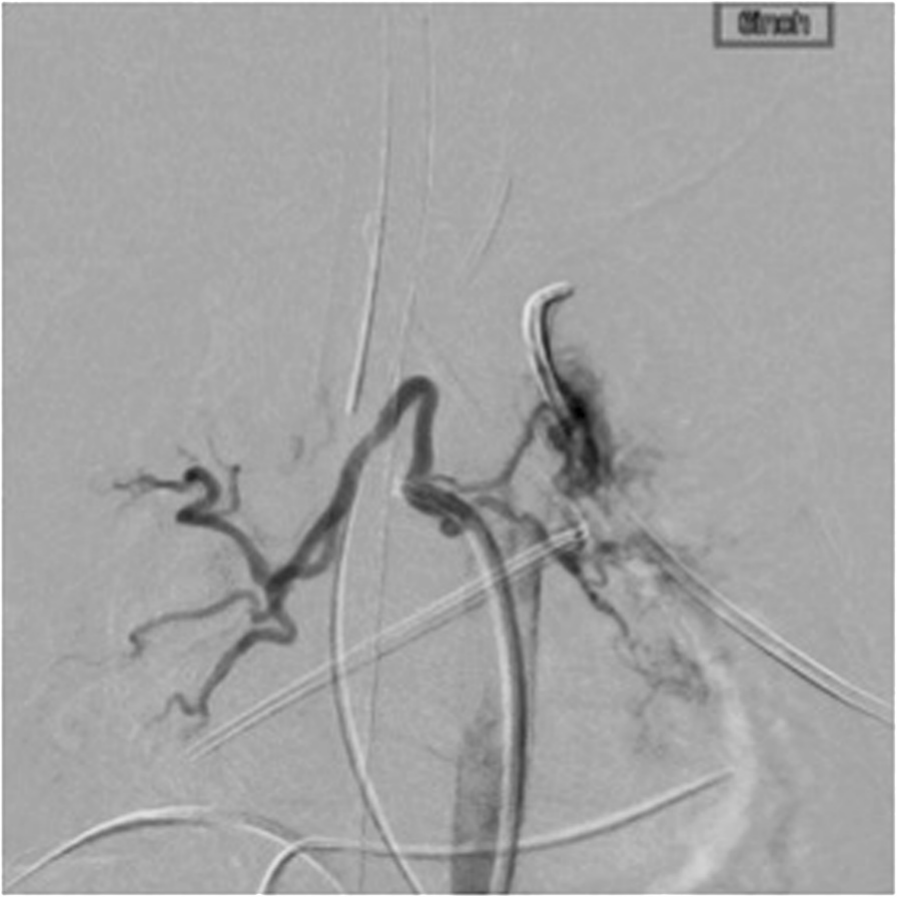
An angiography image showing two aortopulmonary arteries connecting the descending aorta and the right pulmonary artery, and one collateral artery connecting the descending aorta and the left pulmonary artery

**Fig. 2:**
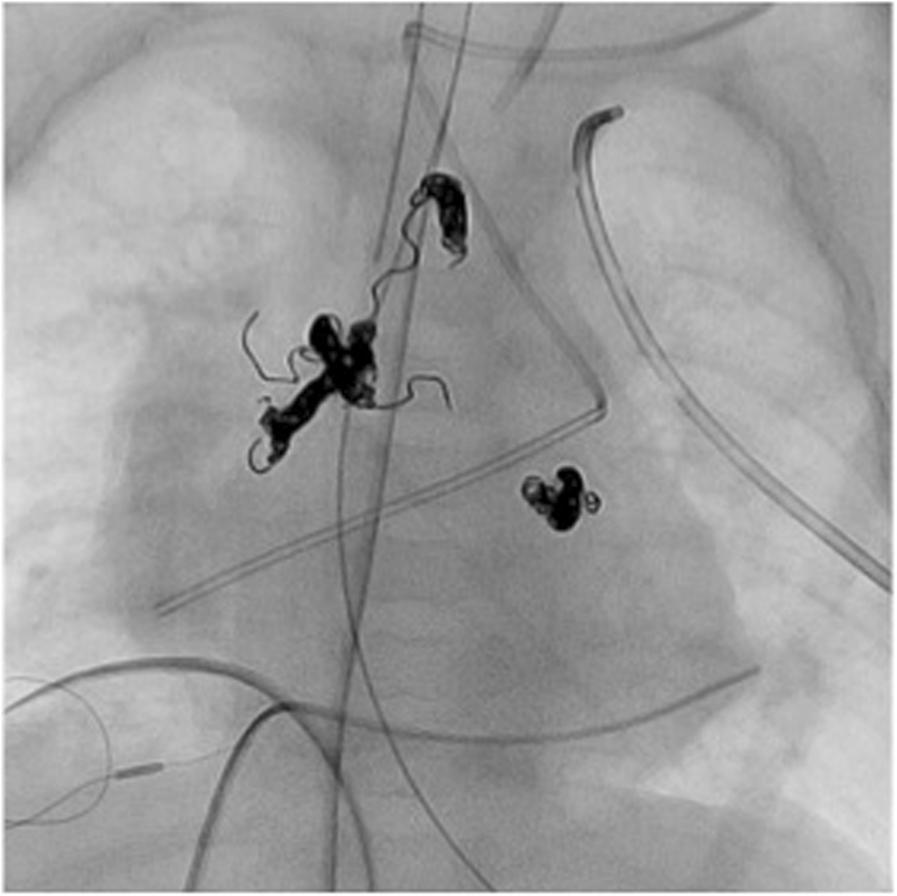
Three abnormal collateral arteries are embolized with 12 Orbit Galaxy detachable coils

A baby girl weighing 1324 g was delivered at 32 weeks 4 days of gestation. She had a very low birth weight and was referred to our hospital with cyanosis. The echocardiographic findings included D-TGA with perimembranous VSD and a restrictive atrial septal defect. After confirming the diagnosis, the ductus arteriosus was kept open with the infusion of prostaglandin E1 until arterial switch operation. Although intensive infant care was provided, at 2 days after birth, she experienced heart failure due to high pulmonary blood flow. She underwent nitrogen inhalation to lower the pulmonary blood flow, and this was maintained until she gained sufficient body weight to undergo arterial switch operation. At 2 months of age, she weighed 2.4 kg and underwent arterial switch operation with VSD closure. The operation was performed as follows: after dissecting around the heart, cardiopulmonary bypass was established with cannulation of the ascending aorta and both the superior and inferior vena cavas, followed by ligation of the ductus arteriosus. Cardiac arrest was achieved with aortic cross-clamping, and glucose-insulin-potassium solution was infused. After we incised the right atrium, we noticed more cardiac return than usual cases of arterial switch operation. The coronary pattern was identified as 1LCx-2R, and the coronary arteries were transferred to each neo-aortic sinus. The VSD was closed through the right atrium with a Gore-Tex patch, and the atrial septal defect was closed with a mattress suture. After the aorta was declamped, the neo-pulmonary trunk was reconstructed with the Lecompte maneuver using an autologous pericardium patch. The duration of cardiopulmonary bypass was 232 min, and aortic cross-clamping time was 187 min. The heart was weaned from cardiopulmonary bypass, and the chest was closed in relatively stable condition. She was transferred to the intensive care unit with infusion of dopamine (5 μg/kg per min) and dobutamine (5 μg/kg per min) and was cared for by skilled medical staff. Suitable postoperative care, including the use of catecholamine and mechanical ventilation, was provided; however, stable blood pressure could not be maintained. Although the diastolic pressure of the femoral artery presented nearly less than 30 mmHg despite the use of adequate dose of inotropic agents, there was no evidence of neo-aortic insufficiency, which is one of the most common reasons for an unstable hemodynamic state. The chest X-rays revealed cardiomegaly; however, we did not realize the presence of aortopulmonary arteries at that time. Additionally, sufficient urine was not being produced, and therefore, she underwent peritoneal dialysis to maintain a proper fluid balance. At 19 days after the operation, an abnormal blood vessel (2 mm in diameter) from the descending aorta to the pulmonary artery was identified on echocardiography. We suspected that this abnormal vessel was responsible for the current heart failure because it was a systemic to pulmonary shunt and had the potential to cause preloading of the left ventricle. We decided to occlude the collateral artery with coils to improve the condition. On arteriography, two aortopulmonary collateral arteries were identified from the descending aorta to the right pulmonary artery and one collateral artery was identified from the descending aorta to the left pulmonary artery (Fig. 1). After confirming the presence of the shunts, the vessels were occluded with 12 Orbit Galaxy detachable coils (DePuy Synthes, Westchester, PA, USA) (Fig. 2). Following this procedure, her hemodynamic condition improved, and she was extubated 6 days after embolization. She was discharged without any complications.

### Discussion

We reported the case of a baby girl treated with coil embolization for abnormal blood flow from the descending aorta to the pulmonary arteries after arterial switch operation. The occurrence of aortopulmonary collateral shunts after transposition of the great arteries is considered rare [3]. One of the possible reasons for overlooking collateral arteries is that the identification of collateral flow may be difficult as it can be obscured by the patent ductus arteriosus. However, large collateral arteries can be problematic immediately after arterial switch operations if they are left untreated [3–6].

In a report by Wernovsky et al., in which neonatal arterial switch operation was the standard surgical treatment for D-TGA, 46 % of patients had abnormal collateral arteries as in our case, and most of the cases had silent symptoms, with only 5 of 119 cases needing coil embolization after arterial switch operation [4]. One of the possible reasons for the development of anomalous vessels is a long period of desaturation [7]. Therefore, we believe that it is crucial to consider the presence of abnormal aortopulmonary shunts in patients with congestive heart failure, who have received nitrogen inhalation to control pulmonary blood flow before operations. In our case, we decided to perform arterial switch operation after the baby gained sufficient body weight. While some babies with low body weight have undergone arterial switch operation in the neonatal period [8], we believe that some institutions need to follow our strategy. The decision to perform arterial switch operations in babies with low body weight depends on the experience and skill of the surgical team. Patients receiving nitrogen inhalation should be carefully checked for collateral vessels after arterial switch operations so that any abnormal vessels can be ligated with surgical techniques or catheter embolization to avoid critical postoperative conditions [9].

## Conclusions

The presence of an aortopulmonary shunt should be considered in patients with unexplained congestive heart failure after arterial switch operation, although the probability of severe symptoms is relatively low.

## Consent

Written informed consent was obtained from the patient for publication of this case report and any accompanying images. A copy of the written consent is available for review by the Editor-in-Chief of this journal.
